# Evidence-Based Nutrition Interventions Improved Adolescents’ Knowledge and Behaviors in Indonesia

**DOI:** 10.3390/nu14091717

**Published:** 2022-04-21

**Authors:** Vanessa M. Oddo, Airin Roshita, Md Tajuddin Khan, Iwan Ariawan, Luh Ade Ari Wiradnyani, Suman Chakrabarti, Doddy Izwardy, Jee Hyun Rah

**Affiliations:** 1 Department of Kinesiology and Nutrition, College of Applied Health Sciences, University of Illinois Chicago, Chicago, IL 61612, USA; 2 Nutrition Section, UNICEF Indonesia, Jakarta 12920, Indonesia; aroshita@unicef.org (A.R.); jhrah@unicef.org (J.H.R.); 3 Department of Economics, Spears School of Business, Oklahoma State University, Stillwater, OK 74078, USA; tajuddin.mse@gmail.com; 4 Reconstra Integra Utama, Jakarta 12950, Indonesia; iwan.ariawan@reconstra.com; 5 Southeast Asian Ministers of Education Organization Regional Centre for Food and Nutrition, Pusat Kajian Gizi Regional (PKGR) Universitas Indonesia, Jakarta 13120, Indonesia; awiradnyani@seameo-recfon.org; 6 Department of Global Health, School of Public Health, University of Washington, Seattle, WA 98195, USA; sc428@uw.edu; 7 Agency of Health Research and Development, Ministry of Health, Jakarta 12950, Indonesia; izwardydoddy@gmail.com

**Keywords:** adolescent nutrition, low- and middle-income countries, school-based intervention, weekly iron-folic acid supplementation, social behavior change communication

## Abstract

Adolescence is a nutritionally vulnerable and critical life stage. However, few programs and policies focus on improving adolescent nutrition in Indonesia. To address this gap, we implemented a gender-responsive package of interventions: (1) breakfast and weekly iron-folic acid supplementation (WIFS), (2) a school-based nutrition education program, and (3) a social behavior change communication strategy. We surveyed 514 adolescents at baseline (2019) and endline (2020) in Klaten and Lombok Barat districts in Indonesia. The survey included a knowledge assessment on nutrition, as well as indicators of attitudes and behaviors on diet, physical activity, and WIFS. We employed multivariable linear and logistic regression to test for pre–post intervention differences. Overall knowledge was significantly higher post-intervention (β: 3.3; 95% confidence interval [CI]: 2.6, 3.9). Diet diversity was high at both timepoints, however, at post-intervention there was significantly higher odds of consuming vitamin A-rich fruits and vegetables (Odds Ratio [OR]: 1.5; 95% CI: 1.1, 2.0) and lower odds of consuming sugar-sweetened beverages (OR: 0.4; 95% CI: 0.3, 0.5). Post-intervention, there was higher odds of reporting 60 min of daily physical activity (OR: 2.3; 95% CI: 1.7, 3.2) and WIFS among girls (OR: 6.7; 95% CI: 1.5, 30.9). The package of interventions may be a promising first step to improving adolescent nutrition in Indonesia.

## 1. Introduction

The triple burden of malnutrition is persistent among adolescents in Indonesia. The 2018 National Basic Health Research Survey found that 26% of adolescents aged 13 to 15 years were stunted, 9% were thin, and 16% were overweight/obese [1]. In addition, 32% of girls aged 15–24 years were anemic [1]. Cross-sectional comparisons of countries show that the prevalence of overweight/obesity in Indonesia is amongst the highest in the East Asia and Pacific region [2]. This is attributed to the changes in dietary intake patterns (i.e., increased consumption of unhealthy foods) and a decrease in physical activity, associated with industrialization and urbanization [3]. Targeted and evidence-based interventions, programs, and policies are warranted to address the evolving nutritional challenges that adolescents face in Indonesia.

A few prior reviews have assessed the effectiveness of nutrition-specific programs for adolescent nutrition [3,4,5,6,7,8,9]. Salam and colleagues’ review highlights the paucity of empirical evidence on what works with regard to adolescent nutrition delivery, but suggested that micronutrient supplementation among adolescents (predominantly females) can significantly decrease anemia prevalence [4,7]. The Salam et al. review also reported that school-based delivery was more effective than community-based initiatives, both for supplementation, as well as nutrition promotion. Lassi and colleagues also highlight the paucity of rigorous evidence testing interventions among adolescent populations [5]. However, similarly, the authors conclude that iron folic acid (IFA) and multiple micronutrient supplements can decrease anemia prevalence among this population. This review also found some evidence supporting strategies for obesity prevention (e.g., diet counseling, physical activity). Verstraeten et al. reviewed school-based obesity interventions in low- and middle-income countries (LMIC) among both children and adolescents and reported that such interventions can increase preferences for healthy foods [8]. However, a review of randomized trials on the effectiveness of obesity prevention interventions for adolescents, mostly from high-income settings, report mixed findings; nevertheless, interventions that combined diet with physical activity had the most promise for obesity prevention [6]. A review of the evidence from Indonesia found that delivering IFA at-scale and providing complementary diet counseling and nutrition education has the potential to improve the nutritional status of adolescents in this context [3]. Additionally, a recent review of interventions that delivered nutrition education on malnutrition among adolescents reported that education tended to improve knowledge, attitudes, and practices, both through school-based or community-based delivery platforms [10].

Despite the aforementioned literature, there is limited operational research and a need to build the evidence-base on multi-sectoral initiatives that specifically target adolescents. While programs delivering weekly IFA supplementation (WIFS) to adolescent girls have been implemented in urban areas of Indonesia [11] and in India [12], to our knowledge studies have yet to evaluate an intervention that integrates WIFS, nutrition education and social behavior change communication (SBCC) in the region. In response, UNICEF Indonesia, in partnership with the government, designed and implemented an evidence-based gender-responsive package of interventions for adolescent nutrition, based on a series of qualitative and quantitative studies and in consultation with multi-sectoral stakeholders, including the government and civil society organizations at the national and sub-national levels. The program, which aimed to improve adolescents’ knowledge, attitudes, and behaviors related to healthy eating and physical activity, was piloted in two districts (Lombok Barat and Klaten) for 15 months between 2019 and 2020; it consisted of three nutrition-specific interventions: (1) home-brought breakfast and WIFS for girls to control and prevent anemia, (2) a nutrition education program that was incorporated into the school curriculum, and (3) a SBCC strategy that included school mobilization, capacity building of key stakeholders (e.g., adolescent peer support groups, health and non-health service providers), and a media campaign. The objective of this study was to assess the effect of the package of interventions on nutrition knowledge, attitudes, and diet and physical activity behaviors among adolescents in Indonesia.

## 2. Methods

### 2.1. Study Context

The study was conducted in Klaten and Lombok Barat districts. Klaten is an urban area and one of the most densely populated municipalities in Central Java Province, with a total population of 1.3 million [13]. Lombok Barat is a more rural area and one of 10 municipalities in West Nusa Tenggara Province, with a total population around 720,000 [14]. Adolescents residing in Klaten generally have a higher socioeconomic status compared to those residing in Lombok Barat [15]. The two districts were selected in consultation with the national and local governments, based on adolescent nutritional status, cultural context, and infrastructure.

### 2.2. Intervention Components

The intervention included three components (see Supplemental Table S1). First, schools in the two selected pilot districts allocated one day per week for adolescent girls to consume IFA supplements (provided by the school) together with breakfast, which is brought from home. Second, along with the IFA distribution, the intervention piloted a weekly 30 min learning session, facilitated by teachers, to generate awareness on nutrition and other relevant topics. UNICEF, in collaboration with the government, developed the multi-sectoral interactive learning materials, which consisted of 36 didactic sessions that covered different topics on nutrition, health, sexual and reproductive health, personal hygiene and sanitation, non-communicable diseases, addictive substances, mental health, and violence/injury. Third, the intervention included SBCC activities, which were designed to involve and empower adolescents in disseminating and promoting key SBCC messages, including prevention of anemia (e.g., take iron/IFA supplements), healthy dietary practices (e.g., choose fresh foods over processed foods), and physical activities (e.g., get 60 min of physical activity every day).

The study was approved by Oxford Policy Management Limited’s internal Ethics Review Committee Study for the baseline survey (March 2019, No. A3347) and Atmajaya University Ethics Review Committee for the endline survey (November 2020, No. 1241A/III/LPPM.PM.10.05/11/2020). Both students and parents provided oral consent prior to participation.

### 2.3. Study Design, Sampling, and Population

Using a pre-post design, we aimed to evaluate the changes in knowledge, attitudes, and behaviors among adolescents after 15 months of the intervention. We employed a stratified random sampling approach. First, schools participating in the national WIFS program were randomly selected for participation (N = 60); this included 48 public junior and 12 senior high schools. Second, a computer-assisted personal interview program (CAPI) was used to randomly select 12 students in Grades 7 and 10 from each school, as we aimed to interview 9 students in each school, and an additional 3 students were sampled as replacements to account for students’ refusal to participate. At baseline, we had a response rate of 98% and successfully recruited 9 students in 60 schools (N = 540 adolescents); this high response rate was achieved through high levels of school attendance and low respondent burden. With a sample of 540 adolescents, a priori estimates suggested a minimal detectable effect of 5–12 percentage points. We assumed a 20% drop out rate, and thus, needed a minimum of 432 participants at endline. We retained a sample size of 514 students at endline (95% of the original sample).

The study population included school-going adolescents aged 12–18 years, in Klaten and Lombok Barat (Table 1). At endline, adolescents were 15.0 years old (95% Confidence Interval: 14.7, 15.4) on average, and 55.8% (95% CI: 49.7%, 60.2%) were female. About one-fifth (20.2% 95% CI: 16.0%, 25.1%) lived in households with low socioeconomic status. A large majority of adolescents used social media (98.8%; 95% CI: 97.6%, 99.4%) and 8.5% of the sample worked for pay (95% CI: 5.9%, 12.0%).

### 2.4. Data Collection

Standardized survey modules (described below and detailed in Supplemental File S1) were developed in English and then translated into the Indonesian language. Modules on dietary intake (FFQ and 24-h recall), physical activity and socio-demographic questions were validated during a 2017 survey conducted in the same districts [16]. Tools and protocols were pre-tested in both districts. The same questions were asked at baseline and endline.

Field staff training included didactic and mock peer-interview components, as well as practice interviews with respondents. Baseline data were collected in-person by the trained field staff between April and May 2019 through a CATI approach. The completion of the questionnaires took ~60 min. Throughout data collection, several data quality assurance mechanisms were implemented (e.g., live data checks, daily uploads of data, a data collection monitoring dashboard, close supervision of survey teams). At baseline, supervisors visited all data collection teams and provided feedback on in-person interviewing during the first two days of data collection and the district coordinators randomly visited data collection teams throughout the duration of the survey to check the quality of interviews conducted and data entry. Endline data were collected via telephone and online between October and November 2020, due to the ongoing COVID-19 pandemic. Accordingly, supervisors reviewed 10% of the phone interviews conducted for each enumerator.

### 2.5. Description of Key Variables

Students self-reported demographic information (e.g., age, gender, socioeconomic status) and responded to questions on nutrition knowledge, healthy eating attitudes and practices, diet, and physical activity. Questions on weekly IFA consumption were included for adolescent girls. Questions on nutrition knowledge were self-administered. All other questions were interviewer-administered.

Nutrition knowledge. The knowledge assessment included 31 multiple choice questions on information delivered during the intervention (Supplemental File S1). Questions related to healthy eating and general nutrition topics (N = 21), physical activity (N = 3), and anemia (N = 7). For example, adolescents were asked to identify 10 Balanced Nutrition Messages, the recommended duration of physical activity and common causes of anemia. Each question was worth a total of 1 point. Since most of the questions were multiple choice, we scaled the points awarded. For example, if 10 response options were listed and 6 were correct, we awarded 0.17 points for each correct answer and a total of 1 point if the student circled all of the 6 correct answers. Individual questions were then summed to create overall knowledge and domain specific scores.

Healthy eating attitudes and practices. Students were asked a series of statements around healthy eating (e.g., would you like to eat more or less fruits every day) and difficulty of practices (e.g., consuming vegetables at every meal) using a five-category Likert scale. Responses ranged from strongly disagree (=1) to strongly agree (=5), which were dichotomized as “agree” versus “disagree”.

Diet. Diet diversity was assessed using a 24-h recall and scored based on the 2016 Food and Nutrition Technical Assistance guidelines [10]. Adequate diet diversity was defined as consuming ≥ 5 of the following 10 food groups: (1) grains, (2) pulses, (3) nuts and seeds, (4) dairy, (5) meat, fish, and organ meat, (6) eggs, (7) vitamin A-rich fruits and vegetables, (8) dark green leafy vegetables, (9) other fruits, and (10) other vegetables. Pulses, nuts, and seeds were collected as part of the same category, so the maximum score was 9 versus the typical 10 groups. Additionally, we collected data on the consumption of unhealthy food items (SSBs, fried and factory-made snacks, and sweets) in the seven days before the survey via food frequency questionnaire.

Physical activity. Students were asked to self-report whether they participated in 60 min of activity per day in the last 7 days and self-rate their physical activity (e.g., very active, active, not active).

Weekly Iron Folic Acid Supplementation. As part of the national WIFS program, schools are asked to distribute IFA tablets to girls, weekly. However, the coverage and compliance are thought to be low. We collected student-level information to understand the proportion of girls consuming IFA (ever and weekly) and whether they perceived that taking IFA was inconvenient or dangerous.

### 2.6. Statistical Analysis

Descriptive statistics were estimated as means or percentages (95% Confidence Intervals [CI]). Our primary analyses used multivariable linear and logistic regression to test for pre-post intervention differences in knowledge, healthy eating attitudes, diet, physical activity, and weekly IFA consumption, when adjusting for sex, grade, district and socioeconomic status. In secondary analyses, we tested for pre-post intervention differences in select indicators, using multivariable regression, stratified by gender.

Alpha was set to 0.05. All results presented use an inverse probability weight; we obtained the actual number of children enrolled in each school from the district education office, which allowed us to produce estimates representing the population of all 7th and 10th—grade students in selected schools (see Supplemental File S2). Standard errors were clustered at school-level. Statistical analyses were performed using Stata 16 (StataCorp LP, College Station, TX, USA).

## 3. Results

### 3.1. Descriptive Results

Descriptive statistics are detailed in Table 2 and Table 3. On average, adolescents answered 13.9 (95% CI: 13.2, 14.7) questions correctly at baseline versus 17.2 questions (95% CI: 16.3, 18.0) at endline (Table 2). At baseline, 41.2% (95% CI: 33.3%, 49.6%) of adolescents answered ≥ 15 questions correctly, compared to 65.9% (95% CI: 59.3%, 72.0%) at endline. About half to three-quarters of adolescents had a positive attitude on healthy eating (e.g., perceived that they should be eating more healthfully) at both timepoints. Similarly, 61.7% (95% CI: 56.7%, 66.5%) and 68.6% (95% CI: 63.9%, 73.0%) of adolescents perceived themselves to be physically active at baseline and endline, respectively.

Overall adequate diet diversity was relatively high, with 60.6% (95% CI: 56.2%, 64.8%) and 56.1% (50.4%, 61.7%) of the sample consuming ≥5 of the core food groups at baseline and endline, respectively (Table 3). Correspondingly, the consumption of staple foods (~99%) and animal source foods (~75%) was high at both timepoints. Comparatively, the reported consumption of vitamin A-rich fruits and vegetables (29.5%; 95% CI: 25.4%, 34.0%) was lower at baseline. At endline, 37.9% (95% CI: 31.3%, 45.0%) of adolescents consumed vitamin A-rich fruits and vegetables. The consumption of sugar-sweetened beverages (SSB) (62.9%; 95% CI: 56.3%, 69.1%) and snack foods (90.0%; 95% CI: 87.0%, 92.4%) was high at baseline. Descriptive statistics suggested the consumption of SSBs (39.5%; 95% CI: 33.0%, 46.5%) and snack foods (72.6%; 95% CI:65.5%, 78.7%) was lower post-intervention.

At baseline, only 23.6% (95% CI: 18.9%, 28.9%) of adolescents participated in at least 60 min of activity per day, compared to 41.1% (95% CI: 36.3%, 46.0%) at endline (Table 3). Nearly all (>95%) of the adolescent girls sampled reported ever taking IFA supplements at both timepoints (Table 3). However, at baseline, about half (56.6%; 95% CI: 46.3%, 66.4%) reported consuming tablets at school during the prior week (before COVID-19), compared to 89.0% (95% CI: 82.0%, 93.5%) at endline. One-third of adolescent girls reported that taking weekly IFA tablets was inconvenient (34.5%) and dangerous (26.7%) at baseline; reporting that IFA supplementation was inconvenient (24.7%) and dangerous (14.1%) was lower at endline.

### 3.2. Multivariable Models

Overall trends were similar in models adjusted for students’ sex, grade, district, and socioeconomic groups. In adjusted models, overall knowledge was significantly higher, when comparing baseline to endline (β: 3.3; 95% CI: 2.6, 3.9) (Table 2). Correspondingly, knowledge scores related to healthy eating (β: 2.2; 95% CI: 1.8, 2.7), physical activity (β: 0.6; 95% CI: 0.4, 0.8), and anemia (β: 0.5; 95% CI: 0.2, 0.7) were significantly higher, baseline to endline. Likewise, the odds of correctly answering 15+ questions was significantly higher at endline, compared to baseline, in adjusted models (Odds Ratio [OR]: 3.3; 95% CI: 2.4, 4.6). Adjusted models suggested that healthy eating attitudes did not change, over time. However, the odds of perceiving that one was physically “active” was 40% higher at endline versus baseline (OR: 1.4; 95% CI: 1.0, 1.9).

The odds of meeting adequate diet diversity was not significantly different in adjusted models (OR: 0.8; 95% CI: 0.6, 1.1) (Table 3). However, there was a significantly higher odds of consuming vitamin A-rich fruits and vegetables (OR: 1.5; 95% CI: 1.1, 2.0), when comparing baseline to endline. We also observed 60% lower odds of SSB consumption (OR: 0.4; 95% CI: 0.3, 0.5) and 70% lower odds of snack food consumption (OR: 0.3; 95% CI: 0.2, 0.4), pre-to post-intervention.

Comparing baseline to endline, there was higher odds of daily physical activity in adjusted models (OR: 2.3; 95% CI: 1.7, 3.2). There was also higher odds of reporting *ever* taking IFA (OR: 6.7; 95% CI: 1.5, 30.9) and consuming IFA tablets at school (OR: 7.0; 95% CI: 4.0, 12.2) among adolescent girls. Likewise, there was higher odds of perceiving that WIFS was not inconvenient (OR: 1.6; 95% CI: 1.0, 2.5) and not dangerous (OR: 2.3; 95% CI: 1.4, 3.8) when comparing baseline to endline.

### 3.3. Secondary Analysis

Similar to our primary results, overall knowledge was significantly higher, when comparing baseline to endline, among both boys (β*:* 2.3; 95% CI: 1.3, 3.3) and girls (β: 4.1; 95% CI: 3.2, 4.9) (Supplemental Table S2). However, among boys, there was a significantly lower odds of meeting adequate diet diversity (OR: 0.7; 95% CI: 0.4, 1.0) over time. Comparing baseline to endline, there was a significantly higher odds of participating in 60 min of daily activity among both boys (OR: 2.1; 95% CI: 1.4, 3.3) and girls (OR: 2.7; 95% CI: 1.7, 4.2).

## 4. Discussion

The objective of this study was to test the effect of a package of three nutrition-specific interventions on nutrition knowledge, attitudes, and diet and physical activity behaviors. The package of interventions was associated with greater knowledge related to healthy eating, physical activity, and anemia. We also observed some changes in healthy eating and physical activity behaviors. Although we did not observe differences between baseline and endline in overall diet diversity, we did observe a significantly higher consumption of vitamin A-rich fruits and vegetables and a significantly lower consumption of unhealthy foods (SSBs and snacks). There was also a significantly higher odds of reporting 60 min of daily physical activity and IFA consumption.

Our baseline findings suggested that there was potential to improve nutrition knowledge and healthy behaviors among Indonesian adolescents. For example, at baseline, adolescents correctly responded to less than half of the questions related to nutrition. Moreover, adolescents widely consumed energy-dense foods, only 24% of students participated in daily physical activity, and the consumption of IFA was irregular, as only 57% reported taking IFA tablets during the prior week at baseline, despite a national program. These baseline estimates are generally consistent with prior reports that Indonesian adolescents have limited knowledge on nutrition [17,18] and data from the Indonesian Family Life Survey, which found that three-quarters of children and adolescents aged 6–18 years consumed instant noodles during the last week and that two-thirds regularly consumed fried snacks, fast food and soda [1,19]. Qualitative research has also found that Indonesian adolescents often eat away from home and purchased food from vendors [14,15]. Moreover, lower levels of baseline physical activity are consistent with prior studies suggesting that adolescents in Indonesia are sedentary [15,20,21].

Importantly, our findings are generally consistent with prior studies that suggest a package of interventions and multi-sectoral coordination is needed to improve the nutritional status of adolescents [3,5,11,22]. Our findings are also consistent with studies suggesting that education and SBCC, guided by behavior change frameworks, as part of a package of interventions, can improve knowledge and some healthy nutrition- and physical activity related behaviors among adolescents [8,20,23,24,25,26] and with one study suggesting that integrating nutrition education into the curriculum delivered by teachers/school staff is an effective delivery platform [8]. Additionally, our findings align with a recent systematic review that suggested that nutrition education related to malnutrition improved knowledge among adolescents [10]; however, most of those studies were conducted in developed countries. Two prior studies, conducted outside Southeast Asia, also find that a package of interventions was associated with higher IFA consumption and an increase in the knowledge, attitude, and practice among adolescent girls [27,28].

While we saw higher knowledge, physical activity and IFA consumption over time, the effect of the intervention on diet were more modest, and healthy eating attitudes did not change. This may be because diet diversity and positive attitudes towards healthful eating were already relatively high at baseline. A lack of improvement in overall diet diversity may also be due, in part, to higher food prices during the COVID-19 pandemic. Additionally, while we observed significantly lower consumption of unhealthful foods pre- versus post-intervention, consumption of these foods remained high; approximately 40%, 73% and 27% of adolescents reported consuming SSBs, snacks, and sweets within the week prior to the endline survey. While some prior studies have showed increased fruit and vegetable intake among adolescents after receiving nutrition education [29,30], our results suggest that substantial changes in diet and attitudes may require additional approaches beyond education and SBCC (i.e., making the healthful choice the ‘easy’ choice). One prior study suggested that while the students may have positive attitudes on healthy eating, healthy foods are less available at schools, leaving them unable to make healthier choices [21]. This is aligned with evidence from Indonesia suggesting that schools may prioritize selling less healthful foods that are profitable [22]. Moreover, adolescents perceive that food availability at school is a key factor in influencing their consumption [8,14]. Although the implementation strategy for this school-based nutrition program, which included multi-sectoral coordination, was generally effective, a key lesson learned was that school-based nutrition education and SBCC strategies may also need to accompany interventions that also alter the food environment and/or disincentivize (or restrict) the purchase and consumption of unhealthy foods and beverages. Gender-targeted approaches may also be warranted given that the mean number of foods groups consumed was in fact lower post-intervention among boys. Given that lifestyle behaviors and weight-status during adolescence track throughout the life course, early prevention is important in order to avoid the onset of chronic diseases in adulthood [31,32].

Our study has some limitations. First, the pre–post only design does not control for changes that could occur without the program and thus, we cannot make causal inferences about the program’s impact. In particular, the program was halted for a period of time during the large-scale closing of schools during the COVID-19 pandemic. We cannot rule out the possibility that COVID-19 social distancing restrictions or school closures biased these results. Second, relatedly, the timeframe for baseline data collection was constrained by several national holidays and national exams. Given the timing of both baseline and endline data collection, it is plausible that student dietary recall may not reflect typical intake. Third, results do not represent all students in Grade 7 and 10 but instead students in Grades 7 and 10 attending the selected pilot schools in this study. Fourth, information on the household-level characteristics were self-reported by students and were confirmed by parents, but no physical verification was conducted. Fifth, although the longer-term goal of improving adolescents’ knowledge and behaviors is to subsequently improve their nutritional status, we did not collect data on height, weight, or micronutrient status.

## 5. Conclusions

The package of interventions was associated with greater knowledge, overall, and changes in physical activity and IFA supplementation behaviors. We observed modest changes in diet. Nevertheless, this school-based package of interventions is a promising first step to increasing stakeholders’ awareness around the urgent need for nutrition interventions among school-going adolescents and ultimately improving adolescent nutrition in Indonesia. In turn, the government will scale-up the intervention nationwide, using the national school health program as the delivery platform. To ensure the success and longer-term sustainability of the program nationwide, advocacy and large-scale capacity building initiatives will continue. Additionally, intensive efforts will be made to adapt this gender-responsive adolescent nutrition program for out-of-school adolescents. There also remains a need for interventions and/or policies that aim to improve the school food environment, which may further support the positive behavior of adolescents toward healthier lifestyles. Future research should test the effect of this package of interventions on adolescent nutritional status.

## Figures and Tables

**Table 1:** Selected demographic characteristics ^1^.

	Mean or % (95% Confidence Interval)	
	Baseline	Endline
Mean Age (years)	13.4 (13.0, 13.8)	15.0 (14.7, 15.4)
Female	55.0% (49.7%, 60.2%)	55.0% (49.7%, 60.2%)
Mean Household size	4.4 (4.3, 4.5)	4.4 (4.3, 4.5)
Low Socioeconomic Status ^2^	19.0% (15.0%, 23.8%)	20.2% (16.0%, 25.1%)
Use social media ^3^	83.6% (78.5%, 87.7%)	98.8% (97.6%, 99.4%)
Paid work in last 7-days	14.8% (11.1%, 19.5%)	8.5% (5.9%, 12.0%)

^1^ Estimated using sampling weights with school-level clustered standard errors. N = 514. ^2^ Total assets are estimated using the DHS wealth index, which is a measure of ownership of selected assets (e.g., televisions and bicycles; materials used for housing construction; and types of water access). ^3^ The proportion of students that have access to at least one social media account (e.g., Facebook, WhatsApp).

**Table 2:** Knowledge assessment and healthy eating attitudes among adolescents ^1^.

	Baseline Mean or % (95% CI)	Endline Mean or % (95% CI)	*β* (95% CI) ^2^	OR (95% CI) ^2^
Knowledge Scores				
Mean Overall (N = 31 Q) ^3^	13.9 (13.2, 14.7)	17.2 (16.3, 18.0)	3.3 (2.6, 3.9) *	
Mean Healthy Eating and General Nutrition (N = 21 Q)	9.5 (9.0, 10.0)	11.7 (11.1,12.3)	2.2 (1.8, 2.7) *	
Mean Physical Activity (N = 3 Q)	1.2 (1.1, 1.3)	1.7 (1.6, 1.8)	0.6 (0.4, 0.8) *	
Mean Anemia (N = 7 Q)	3.2 (3.0, 3.4)	3.8 (3.6, 3.9)	0.5 (0.2, 0.7) *	
≥15 Questions Correct	41.2% (33.3%, 49.6%)	65.9% (59.3%, 72.0%)		3.3 (2.4, 4.6) *
Attitudes				
Should eat more fruits ^4^	76.6% (72.2%, 80.5%)	74.8% (70.3%, 78.9%)		0.9 (0.7, 1.3)
Should eat more vegetables ^4^	77.7% (73.4%, 81.4%)	77.0% (72.2%, 81.2%)		1.0 (0.7, 1.4)
Should eat less fried foods ^4^	56.3% (52.4%, 60.1%)	58.8% (53.7%, 63.8%)		1.1 (0.8, 1.5)
No difficulty consuming vegetables at every meal ^5^	61.7% (54.6%, 68.3%)	65.3% (60.1%, 70.1%)		1.2 (0.9, 1.5)
No difficulty consuming a diverse diet ^5^	51.1% (46.2%, 55.9%)	53.4% (46.6%, 60.0%)		1.1 (0.8, 1.4)
No difficulty choosing water over sweetened beverages ^5^	48.8% (43.2%, 54.5%)	49.6% (44.8%, 54.5%)		1.0 (0.8, 1.4)
No difficulty reducing the consumption of fried foods ^5^	55.5% (49.5%, 61.4%)	60.6% (55.0%, 65.8%)		1.3 (0.9, 1.7)
No difficulty reducing the consumption of salty food ^5^	46.7% (42.0%, 51.5%)	43.0% (36.9%, 49.2%)		0.9 (0.6, 1.1)
No difficulty reducing the consumption of noodles ^5^	34.3% (30.0%, 38.8%)	36.5% (31.8%, 41.5%)		1.1 (0.8, 1.5)
Rates own physical activity as “active”	61.7% (56.7%, 66.5%)	68.6% (63.9%, 73.0%)		1.4 (1.0, 1.9) *

CI = confidence interval; OR = odds ratio; Q = questions. ^1^ Estimated using sampling weights with school-level clustered standard errors. N = 514. ^2^ Beta or Odds Ratio comparing baseline to endline, adjusted for students’ sex, grade, district, and socioeconomic groups. ^3^ Each question was worth 1-point and correct responses were scaled for multiple choice responses. For example, if the question had 3 correct answers the student received 0.33-points for each correct response. ^4^ Queries student as to desire to eat more/less every day than currently. Responses were recorded on a Likert scale of 1 to 5, where 1 represents strongly disagree and 5 represents strongly agree. Strongly agree/Agree = 1, else = 0. ^5^ Queries student as to difficultly following/practicing in daily life? Responses were recorded on a Likert scale of 1 to 5 where 1 represents strongly disagree and 5 represents strongly agree. Strongly disagree/Disagree = 1, else = 0. * *p* < 0.05.

**Table 3:** Diet and physical activity behaviors among adolescents ^1^.

	Baseline Mean or % (95% CI)	Endline Mean or % (95% CI)	OR (95% CI) ^2^
Overall Diet ^3^			
Met adequate dietary diversity	60.6% (56.2%, 64.8%)	56.1% (50.4%, 61.7%)	0.8 (0.6, 1.1)
Core Food Groups ^3^			
Staples (e.g., grains, roots, tubers)	99.7% (98.0%, 100.0%)	99.3% (97.2%, 99.8%)	0.4 (0.0, 5.1)
Nuts and seeds	72.2% (67.7%, 76.2%)	66.0% (61.3%, 70.4%)	0.8 (0.6, 1.0)
Dairy	11.0% (8.4%, 14.2%)	7.9% (5.4%, 11.5%)	0.7 (0.4, 1.2)
Meat, poultry, fish	75.7% (71.8%, 79.3%)	72.0% (66.5%, 76.9%)	0.8 (0.6, 1.1)
Eggs	48.2% (42.1%, 54.3%)	50.6% (45.6%, 55.6%)	1.1 (0.8, 1.5)
Dark green leafy veg	58.1% (52.9%, 63.2%)	64.7% (59.4%, 69.7%)	1.3 (1.0, 1.8)
Vitamin A-rich fruit/veg	29.5% (25.4%, 34.0%)	37.9% (31.3%, 45.0%)	1.5 (1.1, 2.0) *
Other vegetables	54.7% (49.7%, 59.5%)	50.5% (44.6%, 56.3%)	0.8 (0.6, 1.1)
Other fruits	35.3% (29.5%, 41.5%)	26.4% (20.9%, 32.9%)	0.7 (0.5, 0.9) *
Unhealthy Foods ^4^			
SSBs	62.9% (56.3%, 69.1%)	39.5% (33.0%, 46.5%)	0.4 (0.3, 0.5) *
Snacks	90.0% (87.0%, 92.4%)	72.6% (65.5%, 78.7%)	0.3 (0.2, 0.4) *
Sweets	22.9% (17.7%, 29.0%)	27.4% (21.8%, 33.8%)	1.3 (0.9, 1.8)
Physical Activity			
Physical activity for 60 min/day in the prior 7 days	23.6% (18.9%, 28.9%)	41.1% (36.3%, 46.0%)	2.3 (1.7, 3.2) *
IFA Supplementation Among Females			
*Ever* taken IFA tablets	94.6% (89.4%, 97.3%)	99.1% (96.4%, 99.8%)	6.7 (1.5, 30.9) *
Took IFA tablets/wk before school closed due to pandemic	56.6% (46.3%, 66.4%)	89.0% (82.0%, 93.5%)	7.0 (4.0, 12.2) *
Taking IFA weekly is NOT inconvenient	65.5% (56.9%, 73.2%)	75.3% (69.2%, 80.5%)	1.6 (1.0, 2.5) *
Taking IFA weekly is NOT dangerous	73.3% (65.9%, 79.5%)	85.9% (80.2%, 90.2%)	2.3 (1.4, 3.8) *

CI = confidence interval; IFA = iron and folic acid; OR = odds ratio. ^1^ Estimated using sampling weights with school-level clustered standard errors. N = 514 for diet/physical activity. N = 273 for IFA. ^2^ Odds Ratio comparing endline to baseline adjusted for students’ sex, grade, district socioeconomic groups. ^3^ Based on 24-h recall. ^4^ Assessed by FFQ in the seven days preceding the survey. * *p* < 0.05.

## Data Availability

Data are available upon request.

## Supplementary Materials

The following supporting information can be downloaded at: https://www.mdpi.com/article/10.3390/nu14091717/s1, We include the description of the intervention, relevant questionnaires, analysis by gender, and a description of the survey weights in the supplemental file: Table S1: Description of Intervention Components and Key Outcomes, Table S2. Diet and Physical Activity Attitudes and Behaviors Among Adolescents, by Gender, File S1. Selected Survey Questions, File S2. Description of Inverse Probability Survey Weights.
