# Supplementary material for: Evidence-Based Nutrition Interventions Improved Adolescents’ Knowledge and Behaviors in Indonesia
Source: Nutrients. 2022 Apr 21;14(9):1717. doi: 10.3390/nu14091717 (PMC9102904; doi:10.3390/nu14091717)
Supplement: Supplementary file 1 [file nutrients-14-01717-s001.zip › nutrients-1635064-supplementary.pdf]

Evidence-based nutrition interventions improved adolescents' knowledge and behaviors in Indonesia

Supplemental Table S1: Description of Intervention Components and Key Outcomes

Supplemental Table S2. Diet and Physical Activity Attitudes and Behaviors Among Adolescents, by Gender

Supplemental File S1. Selected Survey Questions

Supplemental File S2. Description of Inverse Probability Survey Weights

Supplemental Table S1: Description of Intervention Components and Key Outcomes[1]

Intervention	Description	Measured Outcomes
School-based Weekly IFA Supplementation	• Distribute IFA supplements weekly to girls at school to consume together with breakfast. UNICEF also supports the Government of Indonesia in improving the relevant data and information system.	• IFA consumption (ever, weekly); perceptions of IFA
School-based Nutrition Education Program	• Developed multi-sectoral interactive learning materials designed to improve the knowledge and attitudes of adolescents related to healthy eating and physical activity.<br>• UNICEF implemented a train-the-trainer model (e.g., they trained school health teams on the learning modules).<br>• Students participated in weekly 30-minute learning sessions, for 36 weeks. Sessions are facilitated by trained teachers, to generate awareness on nutrition and health.	• Primary: Knowledge and attitudes related to nutrition and physical activity<br>• Secondary: Diet practices, Physical activity practices
School-based SBCC	• The SBCC gender-responsive activities include school mobilization, capacity building, community-based advocacy, and a media campaign.<br>• SBCC intervention transmitted information on:<br>o Control and Prevention of Anemia (take iron/IFA supplements; eat iron-rich and fortified foods; eat green, leafy vegetables)<br>o Healthy Dietary Practices (eat five servings (five fistfuls) of vegetables and fruits every day; include a fruit or vegetable with every meal; eat colorful vegetables and fruits; choose fresh foods over processed foods; choose water over sweetened beverages or juices; reduce intake of packaged foods)<br>o Physical Activity (get 60 minutes of physical activity every day – walk more, jog, bike, dance, etc; engage in active sports that you enjoy)	• Primary: Knowledge and attitudes related to nutrition and physical activity<br>• Secondary: Diet practices, Physical activity practices

SBCC = social behavior change communication

[1] The activity menu that can be selected is based on the six SBCC intervention modalities: advocacy, capacity strengthening, school mobilization, community engagement, social mass media, and material development

Supplemental Table S2. Diet and Physical Activity Attitudes and Behaviors Among Adolescents, by Gender[1]

	Boys (N=241)	Girls (N=273)
	β or Odds Ratio (95% CI) [2]	β or Odds Ratio (95% CI) [2]
Mean Overall score (Q=31) [3]	2.3 (1.3, 3.3)*	4.1 (3.2, 4.9)*
Mean Healthy Eating score (Q=21)	1.6 (0.9, 2.3)*	2.8 (2.2, 3.3)*
Mean Physical Activity score (Q=3)	0.3 (0.1, 0.5)*	0.7 (0.5, 0.8)*
Mean Anemia score (Q=7)	0.5 (0.2, 0.7)*	0.6 (0.4, 0.9)*
≥ 15 Questions Correct	2.8 (2.0, 3.9)*	3.9 (2.5, 6.1)*
**Attitudes**		
Should eat more fruits[4]	1.2 (0.7, 2.0)	0.7 (0.5, 1.2)
Should eat more vegetables[4]	1.3 (0.8, 2.1)	0.8 (0.5, 1.3)
Should eat less fried foods[4]	1.2 (0.8, 1.8)	1.1 (0.7, 1.6)
No difficulty consuming vegetables at every meal[5]	1.0 (0.6, 1.5)	1.3 (0.9, 2.0)
No difficulty consuming a diverse diet[5]	1.4 (0.9, 2.1)	0.9 (0.6, 1.3)
No difficulty choosing water over sweetened beverages[5]	1.3 (0.8, 1.9)	0.9 (0.6, 1.3)
No difficulty reducing the consumption of fried foods[5]	1.3 (0.8, 2.0)	1.2 (0.8, 1.8)
No difficulty reducing the consumption of salty food[5]	0.8 (0.5, 1.3)	0.9 (0.6, 1.3)
No difficulty reducing the consumption of instant noodles[5]	0.9 (0.6, 1.4)	1.3 (0.9, 2.0)
**Behaviors**		
Mean number of food groups consumed in prior 24 hours	-0.3 (-0.6, 0.0)*	0.1 (-0.2, 0.4)
Adequate dietary diversity	0.7 (0.4, 1.0)*	1.0 (0.7, 1.5)
Physical activity for 60 minutes/day in the prior 7 days	2.1 (1.4, 3.3)*	2.7 (1.7, 4.2) *

CI = confidence interval; Q = questions

[1] Estimated using sampling weights with school-level clustered standard errors.

[2] Beta or Odds Ratio comparing endline to baseline adjusted for students' grade, district, and socioeconomic groups.

[3] Each question was worth 1-point and correct responses were scaled for multiple choice responses. For example, if the question had 3 correct answers the student received 0.33-points for each correct response.

[4] Queries student as to desire to eat more/less every day than currently. Responses were recorded on a Likert scale of 1 to 5 where 1 represents strongly disagree and 5 represents strongly agree. Strongly agree/Agree=1, else=0.

[5] Queries student as to difficultly following/practicing in daily life? Responses were recorded on a Likert scale of 1 to 5 where 1 represents strongly disagree and 5 represents strongly agree. Strongly disagree/Disagree=1, else=0.

*p <0.05

Supplemental File S1. Selected Survey Questions

<table>
<tr><td colspan="4">SECTION A: Demographics</td></tr>
<tr><th>Question number</th><th>Question and instructions</th><th>Answer codes</th><th>Answer</th></tr>
<tr><td>S_A01.</td><td>What is your name?</td><td colspan="2">|__||__|</td></tr>
<tr><td>S_A02.</td><td>What is your date of birth?</td><td colspan="2">|__||__| DD |__||__| MM |__||__| YY</td></tr>
<tr><td>S_A03.</td><td>Interviewer: Write down sex of respondent</td><td>01= Male
02= Female</td><td>|__|</td></tr>
<tr><td>S_A04.</td><td>How many members live in your household?

A household is a group of people who usually live together in the same dwelling, including family and non-family members, and eat from the same pot of food at least for the last 6 months.</td><td colspan="2">|__||__|</td></tr>
<tr><td>S_A05.</td><td>What is the highest school grade of the head of your household head?

The household head is the person who is responsible to the overall upkeep of the household, including financial decisions.</td><td>01= No formal education/ No education
02= Not completed elementary school
03=Completed elementary school => Skip to Error! Reference source not found.
04=Completed Junior high school => Skip to Error! Reference source not found.
05= Completed senior high school/ vocational school=> Skip to Error! Reference source not found.
06= Completed diploma => Skip to Error! Reference source not found.
07=Completed university/S2/S3 degree => Skip to Error! Reference source not found.
98= Don’t know
99= Refused to answer</td><td>|__|</td></tr>
</table>

<table>
<tr><td colspan="4">SECTION B: Socioeconomic Background</td></tr>
<tr><th>Question number</th><th>Question and instructions</th><th>Answer codes</th><th>Answer</th></tr>
<tr><td>S_A06.</td><td>What is the main material of the floor in your house?

Interviewer: Main refers to 50% of the house.</td><td>01=Natural floor (e.g. earth, sand, dung)
02= Wood/planks
03=Bamboo/palm
04=Polished wood/parquet
05= Ceramic/marble/granite
06=Tile/Tiles/Terrazzo</td><td>|__|</td></tr>
</table>

		07=Cement/Brick<br>97= Other (specify)<br>98= Don't know	
S_A07.	What is the main material of the roof in your house?<br><br>***Interviewer: Main refers to 50% of the house.***	01= Natural roofing- thatch, palm leaf, sod<br>02= Wood sirap<br>03= Bamboo<br>04= Zinc/Spandek<br>05= Abestos<br>06= Tile (ceramic or clay)<br>07= Concrete<br>08= Metal tiles<br>97= Other (specify)<br>98= Don't know	|__|
S_A08.	What is the main material of the walls in your house?<br><br>***Interviewer: Main refers to 50% of the house.***	01= Bamboo<br>02= Wood Stem<br>03= Bamboo matting / woven bamboo<br>04= Wood planks/shingles<br>05= Baked brick<br>97= Other (specify)<br>98= Don't know	|__|
S_A09.	How many rooms in your house are used to sleep in?	|__||__|	
S_A010.	What type of fuel does your household **mainly** use for cooking?<br><br>***Interviewer: Main refers to the fuel that is used most of the time for cooking.***	01= Electricity<br>02=LPG/Natural gas (3kg)<br>03= LPG/ natural gas (5 kg or 12 kg)<br>04= Biogas<br>05= Kerosene<br>06= Coal, Lignite<br>07= Charcoal<br>08= Wood<br>09= Straw/Shrubs/Grass<br>10= Agricultural crop<br>11= Animal dung<br>12= No cooking at home<br>77= Other specify<br>98= Dont know	|__|
S_A011.	What is the main source of drinking water at your home?<br><br>***Interviewer: Main is the one most often used by household members.***	01= Piped into dwelling ➔ skip to S_A013<br>02= Piped into yard/plot ➔ skip to S_A013<br>03= Public tap<br>04= Protected well<br>05= Unprotected well<br>06= Protected spring<br>07= Unprotected Spring<br>08= River/Stream	|__|

		09=Pond/Lake<br>10=Dam<br>11=Rain water<br>12=Tanker truck<br>13=Bottled water<br>14= Refill bottled water<br>77=Other (specify)<br>98- Don’t know	
S_A012.	Where is that water source located?	01= In own dwelling<br>02= In own yard/plot<br>03= Elsewhere<br>98- Don’t know	|__|
S_A013.	What kind of toilet facility do members of your household use?<br><br>***Interviewer: if there are several type of toilets in the house, refer to the one mainly used by student.***	01 =Flush toilet to sewer<br>02 =Flush toilet septic tank<br>03 = Shared/Public toilet<br>04 =Traditional pit latrine<br>05 =Yard/bush/forest<br>06 =River/stream/creek (including hanging toilets)<br>77 =Others (specify)<br>98 =Do not know	|__|
**INTERVIEWER: Please select the functional assets owned by any household member.**			
		S_A014. ....Do you or any member of your household own [Asset]?<br><br>1- Yes<br>2- No => Skipt to next asset<br>98- Dont know	S_A015.<br>Is [Asset] currently functionning?<br><br>1- Yes<br>2- No<br>98- Dont know
A	Electricity	|__|	|__|
B	Radio	|__|	|__|
C	Television	|__|	|__|
D	Refrigerator	|__|	|__|
E	Mobile telephone or smartphone	|__|	|__|
F	Fixed line telephone	|__|	|__|
G	Computer (desktop/laptop)	|__|	|__|
H	Bicycle	|__|	|__|
I	Motorbike	|__|	|__|
J	Motor Car/Truck	|__|	|__|
K	Row boat/Canoe	|__|	|__|
L	Animal-drawn cart	|__|	|__|
M	Motor boat	|__|	|__|
N	Ship	|__|	|__|

S_A016.	Do you or any member of your household own any agricultural land? This can be land used to grow crops, raising animal or grazing animals.	01= Yes<br>02= No<br>98= Do not know	|__|
S_A017.	Do you or any member of your household own any house? This can be the house you live in or any other house.	01= Yes<br>02= No<br>98=Don't know	|__|
S_A018.	Do you or any member of your household own a bank account?	01= Yes<br>02= No<br>98=Don't know	|__|
S_A019.	In the last 7 days, have you done any paid work?<br><br>'Paid work' refers to work for which you receive compensation, in cash or in kind, for work that is done. This can include work done in the home, neighbourhood or school.	01 - Yes<br>02 – No => Skip to **Error! Reference source not found.**<br>98 – Do not know => Skip to **Error! Reference source not found.**<br>99 – Refuse to answer => Skip to **Error! Reference source not found.**	|__|
S_A020.	Do you own social media such as Facebook, Instagram, Twitter or Line?	01= Yes<br>02=No<br>98= Don't know	|__|
		S_A021.<br><br>What social media do you use? (Select all that apply)<br><br>01=Yes<br>02=No => Go to next social media<br>98=Don't know => Go to next social media	S_A022.<br><br>In the last 30 days, how many times a week did you use it?<br><br>01= Never<br>02= One to three days a week<br>03=4 to 6 days a week<br>05= 7 days a week (every day)<br>98= Don't know
S_A023.	Facebook	|__|	|__|
S_A024.	Whatsapp	|__|	|__|
S_A025.	Instagram	|__|	|__|
S_A026.	Line	|__|	|__|
S_A027.	Path	|__|	|__|
S_A028.	Twitter	|__|	|__|
S_A029.	BBM	|__|	|__|
S_A030.	Snapchat	|__|	|__|
S_A031.	Pinterest	|__|	|__|

S_A032.	Other (specify)	|__|	|__|

SECTION C: IFA supplementation

Question number	Question and instructions	Answer codes	Answer
S_C01.	Have you ever taken IFA tablets?	01 - Yes<br>02 – No => Skip to **Error! Reference source not found.**<br>98 - Do not know => Skip to S_C018	|__|
S_C02.	Did you consume IFA tablets at school in the last 7 days?	01 – Yes => Skip to S_C04<br>02 – No<br>98 - Do not know => Skip to S_C04	|__|
S_C03.	Why did you not have IFA tablet at school in the last 7 days? Select all that apply.<br><br>***Interviewer: DO NOT READ OUT OPTIONS.***<br><br>Any other reason?	A= IFA was not distributed at school<br>B=I was absent on the day of distribution<br>C= I did not want to have it<br>D=There was not enough IFA for everyone in the class<br>E= I had IFA outside of school that week<br>F= There was no school on regular IFA distribution day<br>G= Boys do not get IFA at this school<br>X= Other (specify)<br>Z= don't know	|__|
S_C04.	Do you usually have IFA tablets at school every week?	01= Yes<br>02= No<br>98= Don't know	|__|
S_C05.	Do you think taking IFA tablet every week is inconvenient?	01= Yes<br>02= No<br>98= Don't know	|__|
S_C06.	Now, think about the last 4 weeks.<br><br>How many times in the last 4 weeks did you have IFA tablets at school?	01= 1 time<br>02= 2 times<br>03= 3 times<br>04= 4 times<br>05= 5 times or more<br>06= Never<br>77= Other (specify)<br>98= Don't know	|__|
Now, I will read you some statement about the consumption of IFA. From strongly agree to strongly disagree, how much do you agree with the following statements? ***Interviewer: Read the statement exactly as written. Do not probe.***			
S_C07.	Consumption of iron-folic acid tablet on a weekly basis can be harmful to my health.	01=Strongly disagree<br>02= Disagree<br>03= Do not agree nor disagree<br>04= Agree<br>05= Strongly agree	|__|

SECTION D: Life cycle and nutrition

Interviewer: Read the statement exactly as written. Do not probe.

Question number	Question and instructions	Answer codes	Answer
S_D01.	I would like to eat more fruits every day than I currently do	01=Strongly disagree<br>02= Disagree<br>03= Do not agree nor disagree<br>04= Agree<br>05= Strongly agree	|__|
S_D02.	I would like to eat more vegetables every day than I currently do	01=Strongly disagree<br>02= Disagree<br>03= Do not agree nor disagree<br>04= Agree<br>05= Strongly agree	|__|
S_D03.	I would like to eat less fried foods every day than I currently do	01=Strongly disagree<br>02= Disagree<br>03= Do not agree nor disagree<br>04= Agree<br>05= Strongly agree	|__|
S_D04.	Consuming diverse diet with most food groups for every meal is difficult for me	01=Strongly disagree<br>02= Disagree<br>03= Do not agree nor disagree<br>04= Agree<br>05= Strongly agree	|__|
S_D05.	It is difficult for me to consume vegetables at every meal.	01=Strongly disagree<br>02= Disagree<br>03= Do not agree nor disagree<br>04= Agree<br>05= Strongly agree	|__|
S_D06.	It is difficult for me to choose water over sweetened beverages.	01=Strongly disagree<br>02= Disagree<br>03= Do not agree nor disagree<br>04= Agree<br>05= Strongly agree	|__|
S_D07.	It is easy for me to reduce how much fried foods I eat every day.	01=Strongly disagree<br>02= Disagree<br>03= Do not agree nor disagree<br>04= Agree<br>05= Strongly agree	|__|
S_D08.	Reducing the consumption of salty food is difficult for me.	01=Strongly disagree<br>02= Disagree<br>03= Do not agree nor disagree	|__|

		04= Agree<br>05= Strongly agree	
S_D09.	It is difficult for me to reduce the consumption of instant noodle.	01=Strongly disagree<br>02= Disagree<br>03= Do not agree nor disagree<br>04= Agree<br>05= Strongly agree	|__|

SECTION Ea: 24hr Dietary recall

Now, I would like to ask you about what you ate yesterday.

S_E01.	How many meals did you have yesterday during the day or the night such a breakfast, lunch and dinner?	|__|	
S_E02.	Yesterday, during the day and night, did you eat anything between meals, such a snack as or similar?	01 – Yes<br>02 – No<br>98 – Do not know	|__|
S_E03.	Was yesterday a typical day for you?	01 – Yes<br>02 – No<br>98 – Do not know	|__|

Now, I would like to ask you about what you ate yesterday during the day of the night. Please describe everything you ate yesterday during the day or night, whether at home or outside the home.

A) *Did you eat anything when you first woke up?*
   a. *If **yes**: Please tell me everything you ate at that time. The interviewer should probe: did you eat anything else? UNTIL RESPONDENT SAYS NOTHING ELSE.*
   b. *If **no:** continue to Question B).*

B) *What did you do after that? Did you eat anything at that time?*
   a. *If **yes:** Please tell me everything you ate at that time. Probe: Anything else? Until respondent says nothing else. Repeat question B) above until participant went to sleep until the next day.*
   b. *If respondent mentions mixed dishes like a PORRIDGE, sauce or stew, probe: c) What ingredients were in that **(MIXED DISH)**? Probe: was there anything else in that **(MIXED DISH)**? Until respondent says nothing else.*

Interviewer: DO NOT READ OUT FOOD GROUPS.

	**Question and instructions**	**S_E04. When food group was eaten**					
		Break-fast<br><br>01 - Yes<br>02 – No	Morning snack<br><br>01 - Yes<br>02 – No	Lunch<br><br>01 - Yes<br>02 – No	Afternoon snack<br><br>01 - Yes<br>02 – No	Dinner<br><br>01 - Yes<br>02 – No	Post dinner snack<br><br>01 - Yes<br>02 – No
**Staples**							

A	Nasi dan produk olahan dari beras (lontong, bubur, bihun, tepung beras)/Rice and Rice product	|__|	|__|	|__|	|__|	|__|	|__|
B	Jagung dan olahannya/Corn and corn product	|__|	|__|	|__|	|__|	|__|	|__|
C	Mie, produk olahan gandum dan tepung lainnya (mie, roti, tepung terigu, oatmeal, energen, tepung kanji, tapioka, mokav)/wheat products; other flours	|__|	|__|	|__|	|__|	|__|	|__|
D	Singkong; Kentang; Umbi-umbian dan sumber karbohidrat lain (ubi, talas, sukun, sagu, tepung sagu, dll)/cassava, potato, other tubers and carbohydrate source	|__|	|__|	|__|	|__|	|__|	|__|
**Roots, tubers and vegetables**							
E	Umbi dan sayur kaya vitamin A: Ubi jalar kuning/oranye/merah/ungu, singkong kuning, wortel, labu kuning, bit/Roots and vegetables rich in vitamin A: carrot; pumpkin; sweet potato yellow/oranye/red/purple; beet	|__|	|__|	|__|	|__|	|__|	|__|
F	Sayuran berwarna hijau tua (daun singkong, kangkung, bayam, berbagai sawi hijau, buncis, daun pepaya, brokoli, katuk, daun kelor, daun melinjo, daun ubi jalar, daun bawang, daun labu, daun turi, kenikir, kemanggi, daun lumbu/talas, genjer, daun kacang panjang, daun semanggi, pakis, glandir, dll)/dark green leaf vegetables	|__|	|__|	|__|	|__|	|__|	|__|
G	Sayuran lainnnya (kubis, sawi putih, tauge, terong, rebung, pare, timun, tomat, jamur, kembang kol, nangka muda, petai, jengkol, daun kelor, dll)/ other vegetables	|__|	|__|	|__|	|__|	|__|	|__|
H	Cabai dan sambal buatan rumah/chili	|__|	|__|	|__|	|__|	|__|	|__|
**Fruits**							
I	Buah berwarna oranye/kaya vitamin A (papaya, mangga, blewah/melon kuning/oranye, terong belanda, semangka kuning/merah, naga merah) atau jus dari buah tersebut tanpa gula /Vitamin A rich fruit/orange-color fruits or their juice	|__|	|__|	|__|	|__|	|__|	|__|
J	Buah lainnya (jeruk, duku, belimbing, anggur, rambutan, melon hijau, nanas, pisang, alpukat, kelapa muda, sirsak, srikaya, apel, jambu air, jambu biji, sawo, durian, manggis, naga putih, nangka matang, dll) atau jus dari buah tersebut tanpa gula/ Other fruits or their juice	|__|	|__|	|__|	|__|	|__|	|__|
**Meat**							
K	Daging unggas (ayam, bebek, burung), abon ayam, ceker, kepala ayam / Poultry	|__|	|__|	|__|	|__|	|__|	|__|
L	Daging berwarna merah dan daging lainnya (sapi, kambing, kerbau, kelinci, ular, biawak), abon sapi, cornet/red meat and other meat	|__|	|__|	|__|	|__|	|__|	|__|
M	Daging babi, daging anjing/pork and dog meat.	|__|	|__|	|__|	|__|	|__|	|__|
**Fish**							
N	Ikan dan seafood (mas, tongkol, bandeng, lele, dll; kepiting, cumi, udang, dll)/fish and seafood	|__|	|__|	|__|	|__|	|__|	|__|
O	Ikan asin/salted fish	|__|	|__|	|__|	|__|	|__|	|__|
P	Sarden kalengan/canned fish	|__|	|__|	|__|	|__|	|__|	|__|
**Organ meat and eggs and other animals**							
Q	Jerohan (ampela, hati, paru, usus, dll)/offal	|__|	|__|	|__|	|__|	|__|	|__|
R	Telur (telur ayam, bebek, puyuh, dan lainnya), telur asin/eggs and salted eggs	|__|	|__|	|__|	|__|	|__|	|__|

<table>
<tr><td>S</td><td>Hewan lainnya (serangga, bekicot, keong, ular, biawak, kodok, ulat jati, kelelawar, dll/ other animals (insects, snails, snake, lizard, frog, maggot, bat, etc)</td><td>|__|</td><td>|__|</td><td>|__|</td><td>|__|</td><td>|__|</td><td>|__|</td></tr>
<tr><td colspan="2">Nuts and legumes</td><td></td><td></td><td></td><td></td><td></td><td></td></tr>
<tr><td>T</td><td>Kacang (kacang tanah, kacang almond, selai kacang, kemiri, dll); Kacang kedelai dan olahannya (tempe, tahu, oncom, kembang tahu, susu kedelai); polong: kacang hijau, kacang panjang, kacang merah/hitam/tolo/kapri/koro /legumes, nuts, soy and soy products</td><td>|__|</td><td>|__|</td><td>|__|</td><td>|__|</td><td>|__|</td><td>|__|</td></tr>
<tr><td colspan="2">Milk and dairy</td><td></td><td></td><td></td><td></td><td></td><td></td></tr>
<tr><td>U</td><td>Susu, termasuk susu rendah lemak (Bear Brand, Frisian Flag UHT, HiLo Teen, Tropicana Slim, Diasweet Litesip, WRP, energen) dan produk olahan susu (yoghurt, keju, yakult)/dairy products/milk including skim milk/its products (yogurt, cheese)</td><td>|__|</td><td>|__|</td><td>|__|</td><td>|__|</td><td>|__|</td><td>|__|</td></tr>
<tr><td colspan="2">Oils and fat</td><td></td><td></td><td></td><td></td><td></td><td></td></tr>
<tr><td>V</td><td>Minyak dan lemak (kelapa sawit, minyak kelapa, minyak sayur);Santan (termasuk parutan kelapa tua, serundeng) ; minyak zaitun, mentega, mayonais)/oil and fat</td><td>|__|</td><td>|__|</td><td>|__|</td><td>|__|</td><td>|__|</td><td>|__|</td></tr>
<tr><td colspan="2">Beverages</td><td></td><td></td><td></td><td></td><td></td><td></td></tr>
<tr><td>W</td><td>Minuman manis (es krim, susu kental manis, es campur (tanpa buah), es doger, cendol, cincau, pop ice, marimas, tebu, es kelapa, legen, air aren, siwalan, buavita, minuman kemasan bergula lainnya)/ Teh/kopi pakai gula atau dalam kemasan /sweet beverages / packed tea/coffee with sugar</td><td>|__|</td><td>|__|</td><td>|__|</td><td>|__|</td><td>|__|</td><td>|__|</td></tr>
<tr><td>X</td><td>Teh/kopi tanpa gula / tea/coffee without sugar</td><td>|__|</td><td>|__|</td><td>|__|</td><td>|__|</td><td>|__|</td><td>|__|</td></tr>
<tr><td>Y</td><td>Softdrink (coca cola, fanta, sprite, big cola, 7up)/softdrinks</td><td>|__|</td><td>|__|</td><td>|__|</td><td>|__|</td><td>|__|</td><td>|__|</td></tr>
<tr><td>Z</td><td>Minuman berenergi (Extra Joss, Kuku Bima, Krating Daeng, Hemaviton, M150, Minuman isotonik (pocari, mizone, hydrococo, UC lemon water, UC100)/ energy drinks, isotonic drinks</td><td>|__|</td><td>|__|</td><td>|__|</td><td>|__|</td><td>|__|</td><td>|__|</td></tr>
<tr><td>AA</td><td>Minuman tradisional/herbal (jamu, kiranti/kemasan berisi jamu)/traditional drink</td><td></td><td></td><td></td><td></td><td></td><td></td></tr>
<tr><td>AB</td><td>Alkohol/Alcohol</td><td></td><td></td><td></td><td></td><td></td><td></td></tr>
<tr><td colspan="2">Snacks</td><td></td><td></td><td></td><td></td><td></td><td></td></tr>
<tr><td>AC</td><td>Makanan ringan goreng – yang isinya kebanyakan hanya tepung dan minyak saja (bakwan, ketan, sosis goreng, chicken nugget, cireng, lanting, tape goreng, risoles, bakso goreng), keripik/kerupuk, macaroni ngehe, jamur goreng, onion ring (bawang), dll /fried snack, deep fried crackers</td><td>|__|</td><td>|__|</td><td>|__|</td><td>|__|</td><td>|__|</td><td>|__|</td></tr>
<tr><td>AD</td><td>Makanan ringan olahan pabrik (ciki, citato, krakers, sosis jadi (so nice), kacang atom, mie kremes, popcorn, dll)/factory snack</td><td>|__|</td><td>|__|</td><td>|__|</td><td>|__|</td><td>|__|</td><td>|__|</td></tr>
<tr><td>AE</td><td>Makanan cepat saji (ayam goreng atau burger kaki lima, pizza, KFC, McD, dll) /fast food</td><td>|__|</td><td>|__|</td><td>|__|</td><td>|__|</td><td>|__|</td><td>|__|</td></tr>
<tr><td></td><td>Sweet foods</td><td></td><td></td><td></td><td></td><td></td><td></td></tr>
<tr><td>AF</td><td>Makanan manis (roti manis, donat, brownies, kue/cake, biskuit, wafer, coklat, permen, madu, gula, kecap manis, agar-agar, pudding, tape singkong/peuyeum, tape ketan, martabak manis, cereal)/sweet food and honey</td><td>|__|</td><td>|__|</td><td>|__|</td><td>|__|</td><td>|__|</td><td>|__|</td></tr>
</table>

0	Nothing	|__|	|__|	|__|	|__|	|__|	|__|

SECTION Eb: Food frequency recall

1. Ask if respondent ate these food items in the past 7 days
2. If respondent ate in the past 7 days, ask how many times in total he/she ate that food (01-95 times) in the past 7 days, not how many days in a week.
3. If respondent did not consume in the past 7 days, fill the frequency with “00” then ask whether respondent have consumed or usually consume the food outside these past 7 days
4. If ever/usually consume, thick 96 = Sometimes
5. If never consume, thick 99 = Never

No	Pangan/Food	Berapa kali /Frequency		96	99	No	Pangan/Food	Berapa kali /Frequency		96	99
**Makanan pokok/Staples**						**Ikan/Fish**					
A	Nasi dan produk olahan dari beras (lontong, bubur, bihun, tepung beras)/Rice and Rice product					N	Ikan dan seafood (mas, tongkol, bandeng, lele, dll; kepiting, cumi, udang, dll)/fish and seafood				
B	Jagung dan olahannya/Corn and corn product					O	Ikan asin/salted fish				
C	Mie, produk olahan gandum dan tepung lainnya (mie, roti, tepung terigu, oatmeal, energen, tepung kanji, tapioka, mokav)/wheat products; other flours					P	Sarden kalengan/canned fish				
D	Singkong; Kentang; Umbi-umbian dan sumber karbohidrat lain (ubi, talas, sukun, sagu, tepung sagu, dll)/cassava, potato, other tubers and carbohydrate source					**Jerohan, telur, dan hewan lain/Organ meat, eggs, and other animal**					
**Umbi dan sayuran/Roots and vegetables**						Q	Jerohan (ampela, hati, paru, usus, dll)/offal				
E	Umbi dan sayur kaya vitamin A: Ubi jalar kuning/oranye/merah/ungu, singkong kuning, wortel, labu kuning, bit/Roots and vegetables rich in vitamin A: carrot; pumpkin; sweet potato yellow/oranye/red/purple; beet					R	Telur (telur ayam, bebek, puyuh, dan lainnya), telur asin/eggs and salted eggs				

F	Sayuran berwarna hijau tua (daun singkong, kangkung, bayam, berbagai sawi hijau, buncis, daun pepaya, brokoli, katuk, daun kelor, daun melinjo, daun ubi jalar, daun bawang, daun labu, daun turi, , kenikir, kemanggi, daun lumbu/talas, genjer, daun kacang panjang, daun semanggi, pakis, glandir, dll)/dark green leaf vegetables					S	Hewan lainnya (serangga, bekicot, keong, ular, biawak, kodok, ulat jati, kelelawar, dll/ other animals (insects, snails, snake, lizard, frog, maggot, bat, etc)				
G	Sayuran lainnnya (kubis, sawi putih, tauge, terong, rebung, pare, timun, tomat, jamur, kembang kol, nangka muda, petai, jengkol, daun kelor, dll)/ other vegetables						**Kacang dan polong/Nuts and legumes**				
H	Cabai dan sambal buatan rumah/chili					T	Kacang (kacang tanah, kacang almond, selai kacang, kemiri, dll); Kacang kedelai dan olahannya (tempe, tahu, oncom, kembang tahu, susu kedelai); polong: kacang hijau, kacang panjang, kacang merah/hitam/tolo/kapri/koro /legumes, nuts, soy and soy products				
**Buah/Fruits**							**Susu dan hasil olahannya/Milk and dairy**				
**I**	Buah berwarna oranye/kaya vitamin A (papaya, mangga, blewah/melon kuning/oranye, terong belanda, semangka kuning/merah, naga merah) atau jus dari buah tersebut tanpa gula /Vitamin A rich fruit/orange-color fruits or their juice					U	Susu, termasuk susu rendah lemak (Bear Brand, Frisian Flag UHT, HiLo Teen, Tropicana Slim, Diasweet Litesip, WRP, energen) dan produk olahan susu (yoghurt, keju, yakult)/dairy products/milk including skim milk/its products (yogurt, cheese)				
**J**	Buah lainnya (jeruk, duku, belimbing, anggur, rambutan, melon hijau, nanas, pisang, alpukat, kelapa muda, sirsak, srikaya, apel, jambu air, jambu biji, sawo, durian, manggis, naga putih, nangka matang, dll) atau jus dari buah tersebut tanpa gula/ Other fruits or their juice						**Minyak dan lemak/Oils and fat**				

**Meat**						**V**	Minyak dan lemak (kelapa sawit, minyak kelapa, minyak sayur);Santan (termasuk parutan kelapa tua, serundeng) ; minyak zaitun, mentega, mayonais)/oil and fat				
**K**	Daging unggas (ayam, bebek, burung), abon ayam, ceker, kepala ayam / Poultry						**Minuman/Beverages**				
**L**	Daging berwarna merah dan daging lainnya (sapi, kambing, kerbau); kelinci, ular, biawak, abon sapi, cornet/red meat and other meat					W	Minuman manis (es krim, susu kental manis, es campur (tanpa buah), es doger, cendol, cincau, pop ice, marimas, tebu, es kelapa, legen, air aren, siwalan, buavita, minuman kemasan bergula lainnya);Teh/kopi pakai gula atau dalam kemasan /sweet beverages / packed tea/coffee with sugar				
**M**	Daging babi, daging anjing/pork and dog meat					X	Teh/kopi tanpa gula / tea/coffee without sugar				
						Y	Softdrink (coca cola, fanta, sprite, big cola, 7up)/sofdrinks				
						Z	Minuman berenergi Extra Joss, Kuku Bima, Krating Daeng, Hemaviton, M150, Minuman isotonik (pocari, mizone, hydrococo, UC lemon water, UC100)/ energy drinks, isotonic drinks				
						AA	Minuman tradisional/herbal (jamu, kiranti/kemasan berisi jamu)/traditional drink				
						AB	Alkohol/Alcohol				
							**Snacks**				
						AC	Makanan ringan goreng – yang isinya kebanyakan hanya tepung dan minyak saja (bakwan, ketan, sosis goreng, chicken nugget, cireng, lanting, tape goreng, risoles, bakso goreng), keripik/kerupuk, macaroni ngehe,				

							jamur goreng, onion ring (bawang), dll /fried snack, deep fried crackers				
						AD	Makanan ringan olahan pabrik (ciki, citato, krakers, sosis jadi (so nice), kacang atom, mie kremes, popcorn, dll)/factory snack				
						AE	Makanan cepat saji (ayam goreng atau burger kaki lima, pizza, KFC, McD, dll) /fast food				
							**Sweet foods**				
						AF	Makanan manis (roti manis, donat, brownies, kue/cake, biskuit, wafer, coklat, permen, madu, gula, kecap manis, agar-agar, pudding, tape singkong/peuyeum, tape ketan, martabak manis, cereal)/sweet food and honey				

SECTION G: Physical Activity

Now, I would like to ask you questions about physical activity and exercising. Physical activity is any activity that increases your heart rate and makes you get out of breath some of the time. Physical activity can be done through sports, playing with friends, walking to school, running, fast walking, biking, dancing, football, menyapu, mengepel, and menyeterika.

Question number	Question and instructions	Answer codes	Answer
Now I would like to ask you some questions about your own physical activity			
S_G01.	From very active to very inactive, how do you rate your own usual physical activity?<br><br>***Interviewer: Do not probe.***	01= Very active => Go to **Error! Reference source not found.**<br>02= Active => Go to **Error! Reference source not found.**<br>03= Not active nor inactive => go to **Error! Reference source not found.**<br>04= Inactive => Go to **Error! Reference source not found.**<br>05= Very inactive => Go to **Error! Reference source not found.**<br>98= Don’t know => Go to **Error! Reference source not found.**	|__|

S_G02.	Did you do any physical activity in the last 7 days for more than 10 minutes?<br><br>Physical activity can be done in sports, playing with friends, or walking to school. Some examples of physical activity are running, fast walking, biking, dancing, football, menyapu, mengepel, and menyeterika.		01-Yes<br>02- No => Skip to **Error! Reference source not found.**<br>98- Don't know => Skip to **Error! Reference source not found.**	|__|
	In the last 7 days, what type of physical activity did you for more than 10 minutes?<br><br>***INTERVIEWER: Probe both out school and out of school.***	S_G03. In the last 7 days, did you do/play [type of exercise] for more than 10 min?<br><br>01- Yes<br>02- No => Skip to next sport<br>98= Don't know => Skip to next sport	S_G04. ....<br>How many times in the last 7 days did you [activity]?<br><br>***Interviewer: Write 98 if student doesn't know***	S_G05. ....<br>How long does a usual session last? (in min)<br><br>***Interviewer: If the student doesn't know, ask about the last session.***<br><br>***Write "998" if student doesn't know.***
	a. Walking- including walking to school or walking to a place far away from your house. This also include walking activity with your class on Fridays.	|__|	|__||__|	|__||__|
	b. Running/Jogging	|__|	|__||__|	|__||__|
	c. Football	|__|	|__||__|	|__||__|
	d. Basketball	|__|	|__||__|	|__||__|
	e. Volley-ball	|__|	|__||__|	|__||__|
	f. Swimming	|__|	|__||__|	|__||__|
	g. Gymnastics	|__|	|__||__|	|__||__|
	h. Dancing	|__|	|__||__|	|__||__|
	i. Cycling	|__|	|__||__|	|__||__|
	j. Martial arts such as karate, Kung-Fu, Taekwondo, Boxing	|__|	|__||__|	|__||__|
	k. Arching	|__|	|__||__|	|__||__|
	l. Badminton	|__|	|__||__|	|__||__|
	m. Household chores such as sweeping, cleaning	|__|	|__||__|	|__||__|
	n. Gardening (at home or at school)	|__|	|__||__|	|__||__|
	o. Cleaning at school	|__|	|__||__|	|__||__|
	p. Ping-pong	|__|	|__||__|	|__||__|
	q. Any other physical activity (specify) for more than 10 min	|__|	|__||__|	|__||__|

Knowledge Assessment

Nutrition

1. Human life is divided into several phases of life (babies-toddlers-children-adolescents-adults-old). Circle all the answers that are considered appropriate about the nutritional and food needs in that phase of life:

    a. We need to consume a variety of foods since we were born
    b. Infants aged under 6 months only need breast milk to grow healthy
    c. The more variety of food we consume, the better
    d. Adolescent girls have the same nutritional needs as adolescent boys
    e. After the age of 6 months, the difference in food for all age groups is in terms of portion/ amount and texture

2. We are encouraged to follow the 10 Balanced Nutrition Messages. Circle all the answers included in the 10 Balanced Nutrition Messages:

    a. Have breakfast every day
    b. Consume sugary drinks so we don't feel hungry easily
    c. Wash the hand with soap and clean running water
    d. Eat a lot of vegetables and fruit
    e. Be grateful and enjoy a variety of foods
    f. Drink milk every day
    g. Limit consumption of carbohydrate-source foods

3. 'My Plate' is a visualization of the principles of balanced nutrition. Which of the following statements is true about 'My Plate'? Circle all the answers that are considered appropriate:

    a. Is a guideline for food portions for one meal
    b. Is a guideline to food consumption for one day
    c. Guideline regarding food consumption, and do not include fluid intake
    d. Is a food consumption guideline for adolescent girls and pregnant women
    e. Has the same meaning as 4 Healthy 5 Perfect
    f. Equipped with suggestions for physical activity

4. Which of the following is the correct guideline regarding 'My Plate'? Circle all the answers that are considered appropriate:

    a. 2/3 of a plate for fruit
    b. 2/3 of the half plate for vegetables
    c. 2/3 of the half plate for staple food
    d. 1/3 of a half plate for side dishes
    e. 2/3 of the half dish for side dishes

5. One of the nutrients the body needs is protein. Which of the statement below is true about protein?

    a. Animal-based protein is less important for the body than plant-based protein
    b. Animal-based and plant-based protein must be consumed in a balanced way
    c. Plant-based contains more complete amino acids
    d. Animal-based protein can cause obesity so it must be avoided
    e. Fish is an excellent example for consumption of plant-based protein

6. Which of the following foods is categorized as a source of plant-based protein? Circle all the answers that are considered appropriate:

    a. Milk
    b. Green beans
    c. Cheese
    d. Tempe
    e. Red beans
    f. Tofu
    g. Long beans
    h. Spinach
    i. Soy
    j. Rice

7. Which of the following foods is categorized as a source of animal-based protein? Circle all the answers that are considered appropriate:

    a. Beef
    b. Tofu
    c. Fresh Shrimp
    d. Scallops
    e. Soy
    f. Mushroom
    g. Milk
    h. Banana
    i. Cheese
    j. Egg

8. Fruits and vegetables have many benefits for our body. Which of the following answers is one of the benefits of consuming fruits and vegetables?

    a. Prevent disease
    b. Gain weight
    c. Energy sources
    d. Replace damaged body cells
    e. Increase muscle mass

9. The recommended portion of vegetables consumed in one day is:

    a. 2-3 servings
    b. 3-4 servings
    c. 1-2 servings
    d. <2 servings
    e. No suggested portions

10. Which of the foods below is the main source of vitamin C?

    a. Guava, orange, papaya
    b. Apples, oranges, carrots
    c. Guava, avocado, eggplant
    d. Orange, spinach, soursop
    e. Carrots, avocados, eggplants

11. Vitamins and minerals are classified as micronutrients. Which of the following statements is true about micronutrients?

    a. Micro nutrients are only contained in fruits and vegetables
    b. Needed in small amounts but need to be consumed in sufficient quantities
    c. Must be consumed in the same amount as carbohydrates and protein
    d. The main food source for micronutrients is rice
    e. Micro nutrients are the main ingredients for producing energy in our body

12. Which of the following answers is correct about the function of calcium?

    a. Manage blood clots when an injury occurs
    b. Formation of bones and teeth
    c. As an antioxidant
    d. Maintain eye health
    e. Produce energy

13. Which of the following answers is correct about the function of iron?

    a. Maintain healthy bones and teeth so that they are not easily broken / brittle
    b. Forms insulin to control blood sugar levels and prevent diabetes
    c. An important component of red blood cells that binds oxygen to be circulated throughout the body
    d. Play a role in muscle activity and blood clotting
    e. Maintaining the acid-base balance/ body pH and osmolarity balance of body fluids

14. Which of the following answers does not belong to one food group:

    a. Rice, corn, noodles, cassava

b. Tofu, green beans, soybean
c. Beef, eggs, kidney beans, shrimp
d. Long beans, kale, bean sprouts, beans
e. Shellfish, beef liver, fish, milk

15. Consumption of sugar, salt and fat must be limited. Which of the following answers is the maximum consumption per person in one day as recommended by the Ministry of Health?

    a. 4 tablespoons of sugar, 1 teaspoon of salt, and 4 tablespoons of oil/ fat
    b. 5 tablespoons of sugar, 1 teaspoon of salt, and 5 tablespoons of oil/ fat
    c. 4 tablespoons of sugar, 1 tablespoon of salt, and 4 tablespoons of oil/ fat
    d. 3 tablespoons of sugar, 1 teaspoon of salt, and 5 tablespoons of oil/ fat
    e. 4 tablespoons of sugar, 1 teaspoon of salt, and 5 tablespoons of oil/ fat

16. Which of the following statements is true about salt, sugar, and fat? Circle all the answers that are considered appropriate:

    a. Some foods, like avocados, have high levels of unsaturated fats that are good for our health
    b. Consumption of too much sugar can cause obesity and diabetes
    c. Fat is not needed by our body because it will increase our weight
    d. Consumption of too much salt can increase the risk of high blood pressure
    e. We do not need to worry about our snacking habits, because the main source of sugar and salt intake is the main food

17. According to the 10 Balanced Nutrition Messages, we are advised to read food labels. Which of the following information is important to consider when looking at labels on food packaging? Circle all the answers that are considered appropriate:

    a. The constituent food ingredients
    b. Nutrition value/ content
    c. Price
    d. Expired date
    e. Packaging size
    f. Suggested serving
    g. Marketing authorization

18. Which of the following statements is true about breakfast?

    a. Not everyone needs breakfast because it depends on individual needs
    b. The main source of carbohydrates in the breakfast menu must be rice
    c. Breakfast provides energy for the brain to work more optimally while studying
    d. Breakfast can cause drowsiness
    e. A healthy breakfast must be accompanied by milk

19. Food and drinks sold without permission in the school canteen are at risk of being contaminated with biological or chemical substances which can endanger the health of students. Which of the following statements is categorized as one of the criteria for a healthy school canteen?

    a. Selling food and drinks with attractive colors
    b. Providing food and drinks at low prices
    c. There are toilets in the school canteen that can be easily accessed by students
    d. Located close to a temporary garbage collection place so food vendors can dispose of garbage easily
    e. There is a place to wash equipment with running water

20. Which of the following answers is true about healthy and safe snacks?

    a. Contains flavor enhancers
    b. Contains preservatives
    c. Not contaminated with physical pollutants such as hair and dirt
    d. Does not contain salt, sugar, and fat
    e. Contains dyes not specifically for food

21. One indicator/ marker of nutritional status for children aged 5-19 years is body mass index according to age (BMI/ age). Which of the following information is not needed to determine BMI/ age?

    a. Gender
    b. Age
    c. Weight
    d. Height
    e. Arm circumference

Physical Activity

1. World Health Organization (WHO) recommends children aged 5-17 years to do physical activities every day. What is the minimum duration of physical activity recommended by WHO for this age group?

    a. 30 minutes per day
    b. 5 minutes per day
    c. 60 minutes per day
    d. 75 minutes per day
    e. 90 minutes per day

2. Regular physical activity provides many health benefits. Which of the following statements is incorrect about physical activity?

    a. Maintaining a balance between incoming energy and release to prevent obesity
    b. Physical activity can only be done with friends
    c. Physical activity does not always have to be sports
    d. Physical activity can be done anywhere and anytime
    e. Physical activity can maintain a smooth blood flow

3. Physical activity for children aged 5-17 years can be done through various activities. Which of the following examples is not included as a physical activity that can be carried out every day (in daily life)?

    a. Choose to walk to school instead of using a motorcycle
    b. Clean the house while dancing
    c. Choose to climb up the stairs instead of using the elevator
    d. Play soccer games on mobile phone
    e. Sweeping the yard while singing

Anaemia

1. One of the most common nutritional problems encountered in adolescents is anaemia. Which of the following statements is considered as one of the causes of adolescents having the risk of anaemia?

    a. Have outdoor activities
    b. Have increased nutritional needs because the body is growing and developing rapidly
    c. Have a habit of doing low physical activity
    d. Consume too much food derived from animal origin
    e. Consume too many sugary drinks

2. What are the common symptoms of anaemia? Circle all the answers that are considered appropriate:

    a. Rapid weight loss
    b. Loss of appetite
    c. Dizzy
    d. Weak
    e. Restless
    f. Difficult to concentrate
    g. Feel tired
    h. Drowsy

3. Adolescent girls have a greater risk of experiencing iron deficiency compared to adolescent boys. One reason is:

a. More adolescent girls experience low blood pressure
b. Adolescent girls have more activities
c. Adolescent boys exercise more often
d. Adolescent boys have better eating habits than teenage girls
e. Adolescent girls lose iron during menstruation

4. Anaemia is a disease that can be prevented. Which of the following statements is not considered a way to prevent anaemia?

   a. Increase physical activity
   b. Consume iron folic acid tablets regularly
   c. Consume food sources rich in iron
   d. Maintain personal hygiene
   e. Practicing balanced nutrition

5. What are some examples of foods that are rich in iron?

   a. Eggplant, corn
   b. Chicken liver, soybean
   c. Bananas, potatoes
   d. Avocados, cabbage
   e. Rice, sweet drink

6. Adolescent girls can experience side effects after consuming iron folic acid tablets. What are the possible side effects? Circle all the answers that are considered appropriate:

   a. High blood pressure
   b. Feeling weak
   c. Nausea
   d. Sleepy
   e. Black stool
   f. Faster heartbeat

7. The following is one of the recommended ways to minimize the side effects of taking iron folic acid tablets:

   a. Take iron folic acid tablets with tea
   b. Take iron folic acid tablets after a meal
   c. Take iron folic acid tablets with coffee
   d. Consume iron folic acid tablets not on a regular basis
   e. Take blood tablets with sweet drinks

Supplemental File S2. Description of Inverse Probability Survey Weights

Student level weights are based on the probability of a student to be sampled within their school.

In our survey, the probability of students to be sampled can be defined as follows:

$$(1)\ p_i = \frac{n_i}{N_i},$$

where n_i is the number of students sampled per school i, N_i is the total number of students in grade 7 or grade 10 present in the school from which the sample is drawn (note that the two are in separate schools), and p_i is the resulting probability of each student to be sampled. As explained above, our sampling approach was to sample 9 students from the list of students present on the day of the survey, i.e. $n_i = 9$. The above probability defined in (1) can be inverted to create weights as follows:

$$(3)\ w_i^{student} = \frac{1}{p_i}.$$

Here, $w_i^{student}$ is the resulting weight for students in school i.

Within each school, all students have the same weights. We rescaled student weights so that they sum up to the actual number of observations in our sample and truncated them at the 80th percentile of the distribution in order to prevent very large weights.
